# Perioperative phase angle decline is associated with postoperative sarcopenia in esophageal cancer: a longitudinal cohort study with internal validation

**DOI:** 10.3389/fnut.2026.1926195

**Published:** 2026-09-04

**Authors:** Yuntao Hao, Yanli Wang, Yiyuan Zhao, Xiugeng Zhou, Shaolei Li, Shi Yan, Nan Wu, Xiao Dong, Liqing Gong, Ziqi Liu, Qi Yu, Zhi Peng, Yu Fang

**Affiliations:** 1 Key Laboratory of Carcinogenesis and Translational Research (Ministry of Education), Department of Clinical Nutrition, Peking University Cancer Hospital and Institute, Beijing, China; 2 Key Laboratory of Carcinogenesis and Translational Research (Ministry of Education), Department of Head and Neck Surgery, Peking University Cancer Hospital and Institute, Beijing, China; 3 Key Laboratory of Carcinogenesis and Translational Research (Ministry of Education), Department of Thoracic Surgery, Peking University Cancer Hospital and Institute, Beijing, China; 4 Key Laboratory of Carcinogenesis and Translational Research (Ministry of Education), Department of Gastrointestinal Oncology, Peking University Cancer Hospital and Institute, Beijing, China

**Keywords:** bioelectrical impedance, esophageal cancer, nutritional assessment, phase angle, phase angle decline, postoperative sarcopenia

## Abstract

**Background and aim:**

Sarcopenia is highly prevalent in patients with esophageal squamous cell carcinoma (ESCC) and is associated with increased postoperative complications, prolonged hospitalization, and reduced survival. While preoperative PhA has been associated with clinical outcomes, the association between perioperative PhA change and postoperative sarcopenia remains unexplored. This study aimed to evaluate whether perioperative phase angle decline (ΔPhA) is associated with postoperative sarcopenia (Sarcopenia_Post). We also assessed the association between preoperative PhA (PhA_Pre) and postoperative outcomes.

**Methods:**

In a prospective longitudinal cohort of 90 patients undergoing esophagectomy for ESCC, phase angle was measured 1 day before and 1 month after surgery. Associations of the primary outcome postoperative sarcopenia with ΔPhA (dichotomized at the cohort mean of 0.32°) were assessed using multivariate logistic regression, adjusting for preoperative sarcopenia and nutritional risk (NRS2002 ≥ 3). Model robustness was confirmed using internal validation approaches including ridge regression, bootstrap validation and random forest benchmarking. Multivariate analyses also assessed associations between PhA_Pre and postoperative outcomes.

**Results:**

A pronounced perioperative decline in PhA (≥0.32°) was independently associated with postoperative sarcopenia after adjusting for preoperative sarcopenia and nutritional risk (adjusted Odds Ratio (aOR) = 4.69, 95% CI: 1.08–20.45, *p* = 0.040). This association was robust, as demonstrated by internal validation techniques. The model demonstrated good discriminatory power (AUC = 0.84, 95% CI: 0.72–0.96). In contrast, the static preoperative phase angle was not significantly associated with postoperative sarcopenia, overall complications, infectious complications, or length of stay in multivariate analyses.

**Conclusion:**

Perioperative phase angle decline is associated with postoperative sarcopenia in patients with ESCC. Given the concurrent timing of assessments, this finding reflects association rather than prediction. The dynamic nature of phase angle change may provide valuable insights into postoperative sarcopenia, and further longitudinal studies with earlier PhA assessments are needed to determine whether perioperative PhA change has predictive utility for subsequent sarcopenia development.

## 1 Introduction

Esophageal cancer is a leading cause of cancer-related mortality worldwide, posing a substantial and growing public health burden (1, 2). Notably, esophageal squamous cell carcinoma (ESCC) accounts for over 85% of global esophageal cancer cases and is particularly dominant in East Asia (3). This histologic subtype is characterized by a unique pathogenesis, tumor biology, and clinical prognosis compared to adenocarcinoma (4). Patients with ESCC are highly susceptible to malnutrition, with reported prevalence ranging from 67% to 85% (5, 6). This frequently presents as sarcopenia, characterized by progressive loss of skeletal muscle mass and function, which is highly prevalent among patients with ESCC and carries important clinical implications (7, 8). In this setting, sarcopenia is closely linked to increased postoperative complications (6), prolonged hospital stays (9), and reduced long-term survival (10). Accordingly, early identification of patients at risk for sarcopenia is critical to guide nutritional and rehabilitative interventions to improve perioperative recovery (11, 12) and survival (13).

Comprehensive nutritional evaluation integrates anthropometric measurements, dietary intake, biochemical indices, and functional assessments (14). Among these, body composition analysis provides critical insights into skeletal muscle mass and cellular health. Phase angle (PhA), derived from bioelectrical impedance analysis (BIA), represents the ratio of reactance to resistance and reflects cell membrane integrity, tissue quality, and hydration status (15). Unlike indirect estimates of body composition, PhA is directly measured and independent of predictive equations. Lower PhA values indicate impaired cellular health, malnutrition, and sarcopenia (16, 17). Studies across various cancer types, including gastrointestinal, head and neck, and lung cancers, have demonstrated that PhA is associated with malnutrition, sarcopenia, disease progression, and survival (18, 19). A recent retrospective cohort study in gastric cancer patients found that sarcopenia predicted poor long-term survival but was not associated with postoperative complications (20), suggesting that the prognostic implications of sarcopenia may differ by cancer type and outcome.

Despite these advances, evidence regarding PhA in the perioperative setting of ESCC remains limited. Previous studies of PhA in oncology have largely focused on prognostic or advanced-cancer populations (21, 22). More recently, PhA has also been investigated in surgical esophageal cancer populations, including in relation to postoperative pneumonia and survival (23). Moreover, these studies often rely on single, baseline PhA measurements, which may fail to capture the impact of perioperative catabolic stress and dynamic changes in cellular integrity (23, 24). We therefore hypothesized that monitoring the decline in PhA throughout the perioperative period (ΔPhA) would be more strongly associated with postoperative sarcopenia than a static preoperative value.

To test this, we conducted a prospective study in Chinese ESCC patients undergoing esophagectomy. Associations between perioperative ΔPhA and postoperative sarcopenia were evaluated using robust statistical techniques. Secondary exploratory analyses examined associations between preoperative PhA and other postoperative outcomes, including complications and length of hospital stay.

By focusing on perioperative PhA dynamics in a surgical ESCC cohort, this study aims to characterize whether changes in PhA are associated with postoperative sarcopenia and to explore their potential clinical relevance for perioperative nutritional assessment and management.

## 2 Materials and methods

### 2.1 Settings and participants

Participants were sampled from the thoracic cancer department of a tertiary cancer hospital in Beijing, China between 2017 and 2022. The inclusion criteria were designed to ensure a homogeneous cohort of patients undergoing curative-intent esophagectomy for ESCC: (a) age ≥18 years; (b) diagnosis of esophageal squamous cell carcinoma, confirmed via pathological examination; (c) scheduled to undergo elective surgery for esophageal cancer within 2 weeks of admission; and (d) voluntary agreement to participate in this study. The exclusion criteria were selected to minimize confounding: (a) presence of other malignancies; (b) cancer recurrence or metastasis, (c) implanted cardiac pacemaker or arterial stent, and (d) cognitive or communication disorders.

The sample size was determined by the available prospective cohort. Initially, 110 patients were enrolled to account for potential exclusions and loss to follow-up. The final analytical cohort included 90 patients, providing 17 events of postoperative sarcopenia and 37 events of postoperative complications, of which 28 were infectious complications. Given the exploratory nature of this prospective cohort and the limited number of postoperative sarcopenia events, model complexity was intentionally restricted and internal validation procedures were applied to reduce overfitting, consistent with recommendations for regression modeling in small datasets (25).

### 2.2 Examinations and measurements

#### 2.2.1 General information and clinical information

In this study, a self-designed questionnaire survey was used to collect the patient’s demographic characteristics and medical history. The questionnaire was designed to capture comprehensive demographic, clinical, and nutritional information. It included: (a) general information (e.g., age, gender, stage of cancer, comorbidities, history of smoking behavior and alcohol consumption, preoperative radiotherapy and chemotherapy); (b) nutritional information before surgery (e.g., BMI, body weight loss, grip strength, calorie intake, protein intake); and (c) clinical information after surgery (e.g., hospital length of stay, clinical complications, energy intake, protein intake). The infectious complications were defined as the complications with Clavien–Dindo grade II or higher, categorized based on therapeutic interventions by clinicians rather than prophylactic antibiotic administration (26). Conditions included pulmonary infection, incision infection, and anastomotic fistula. These data were prospectively collected from electronic medical records from the postoperative stage to discharge and at 1 month after surgery.

#### 2.2.2 Body composition analysis

Body composition data were collected using the InBody 770 multifrequency bioelectrical impedance analyzer (Biospace Co., Ltd., Seoul, South Korea) at an 80 μA current. Intracellular water (ICW), extracellular water (ECW), resistance (R), reactance (Xc), percent body fat (PBF (%)), basal metabolic rate (BMR), arm muscle circumference (AMC), arm circumference (AC), triceps skinfold thickness (TSF), fat-free mass index (FFMI) and fat-free mass (FFM) were recorded. The formulas were as follows:


$$\left(a\right)\,\mathrm{phase}\,\mathrm{angle}\,\left(\mathrm{PhA}\right)=\mathrm{arctangent}\,\left(\mathrm{Xc}/R\right)\times \left(180^{{}^{\circ}}/\pi \right);$$

(b)⁢AMC⁢(cm)=AC⁢(cm)-π×TSF⁢(cm);

$$\left(c\right)\,\text{FFMI}=\mathrm{FFM}\,\left(\mathrm{kg}\right)/\mathrm{height}\,{\left(m\right)}^{2};a\,n\,d$$

(d)⁢ECW/TBW⁢Ratio=ECW÷TBW⁢(27).

The spectral bioelectrical impedance devices used were calibrated daily. Measurements were conducted 1 day before surgery and at the 1-month postoperative follow-up. All assessments were conducted in the morning, with participants instructed to remove all metal-containing objects and empty their bowel and bladder 30 min before the measurements. Participants were also asked to abstain from food or drink intake and vigorous physical activity within 6 h, as well as from alcohol or caffeinated beverages within 48 h before the assessment. During the test, participants removed shoes and socks, stood on the instrument with heels placed on the circular metal electrodes at the base and soles on the front oval electrodes. Body weight was recorded in this standing position. Patients then grasped the handles with thumbs placed on the upper electrodes, while keeping their arms abducted at a 15° angle from the trunk.

#### 2.2.3 Assessment of nutritional status

Nutritional status was comprehensively evaluated, by a trained dietitian using anthropometric, dietary, scoring, and biochemical methods.

Anthropometry: Height and weight were measured using a calibrated stadiometer and digital scale to calculate body mass index (BMI, kg/m^2^). Grip strength (kg) was assessed triplicate on the non-dominant hand using a dynamometer (EH101, purchased from Guangdong Xiangshan Weihua Group Co., Ltd.) with the patient standing and arms neutrally positioned. Three measurements were obtained, and the mean value was used for analysis (28).

Dietary intake: Dietary intake was assessed using three consecutive 24-h dietary recalls, following the Shanghai Clinical Nutrition Model. Energy portions were standardized, with 90 kcal defined as one portion. A trained dietitian calculated the average intake of energy and protein over the 3-day period. Nutrient intake was analyzed using self-developed nutritional software based on the 2009 China Food Composition Table (29).

Nutritional risk screening and malnutrition identification: Nutritional risk was identified using the Nutritional Risk Screening 2002 (NRS2002) tool, with a score ≥ 3 indicating risk (30). Nutritional status was further detailed using the scored patient-generated subjective global assessment (PG-SGA), specific to oncology populations. PG-SGA scores categorized patients as well-nourished (0–3), moderately (4–8), or severely (≥9) malnourished (31).

Sarcopenia diagnostics: Sarcopenia was assessed based on the 2019 AWGS criteria (32). Diagnosis requires: (a) Appendicular Skeletal Muscle Mass Index (ASMI) <7.0 kg/m^2^ for men or <5.7 kg/m^2^ for women; (b) grip strength <28 kg for men or <18 kg for women; and (c) walking speed over 6 m ≤1.0 m/s was also measured. Sarcopenia can be diagnosed by fulfilling the criterion of (a) + (b).

Biochemical tests: After 12 h of fasting, venous blood samples were collected and analyzed within 1 h. Standard laboratory methods were used to measure albumin (ALB), prealbumin (PALB), serum creatinine (Crea), hemoglobin (HGB), and white blood cell (WBC) count using automated biochemical analyzers in the clinical laboratory of the cancer hospital.

### 2.3 Statistical analysis

Data are presented as mean ± standard deviation, median (interquartile range), or number (percentage), as appropriate. All analyses were conducted in R software (version 4.4.0). A two-sided *p* < 0.05 was considered statistically significant. Continuous variables were assessed for normality using Shapiro–Wilk tests and QQ plots. Given the modest sample size and the frequent violation of normality among nutritional biomarkers, skewed variables were compared using the Wilcoxon rank-sum test for independent groups and the Wilcoxon signed-rank test for paired comparisons, consistent with previous perioperative nutritional studies (23). Normally distributed variables were analyzed using parametric equivalents (independent *t*-test for between-group comparisons and paired *t*-test for pre- to postoperative changes). Categorical variables were compared using Pearson’s χ^2^ test or Fisher’s exact test where appropriate, with McNemar’s test for paired categorical variables.

#### 2.3.1 Primary analysis

Univariate logistic regression was first used to assess associations between postoperative sarcopenia (Sarcopenia_Post) and candidate independent variables, including baseline demographic characteristics, clinical parameters, nutritional indices, and body composition measures. Variables with *p* < 0.10 in univariate analyses, together with clinically relevant variables (33), were considered for inclusion in the multivariable model. Potential collinearity was evaluated using Spearman pairwise correlations, with categorical variables numerically encoded, and a correlation of | ρ| > 0.7 for any variable pair was considered indicative of possible collinearity. To assess variable stability and reduce the risk of overfitting, we applied bootstrap resampling with 2000 iterations and ridge regression under both the optimal (λ_min) and conservative (λ_1se) penalization thresholds. Variables with bootstrap coefficient of variation (CV) > 150% were considered unstable, while variables whose ridge regression coefficients were shrunk close to zero were deemed to have minimal independent contribution (34, 35). Based on these complementary approaches, unstable or non-contributory variables were excluded from the final model. This strategy allowed us to quantify variation in coefficient estimates and effect sizes, guiding robust variable selection for the multivariable model.

ΔPhA was defined as PhA_Pre - PhA_Post (postoperative PhA), so that a positive ΔPhA indicates a perioperative decline in PhA. The association between categorized perioperative phase angle decline (ΔPhA_Cat, dichotomized at the cohort mean of 0.32°) and Sarcopenia_Post was then evaluated using multivariable logistic regression. Based on these assessments, the final model included preoperative sarcopenia status (Sarcopenia_Pre) and nutritional risk (NRS2002, dichotomized as ≥3 or <3) as covariates. An extended exploratory model additionally included gender, age, and preoperative PALB to assess potential incremental value beyond the primary model. Results are reported as adjusted odds ratios (aORs) with 95% confidence intervals (CIs). To assess whether the observed association depended on categorization, we additionally analyzed ΔPhA as a continuous variable.

Model performance was assessed using the area under the receiver operating characteristic curve (AUC), a standard measure of discriminative ability in clinical prediction models. Comparisons of AUCs between models evaluated in the same participants were performed using DeLong’s test (36), which accounts for the correlated nature of the ROC curves. Model calibration was evaluated graphically, and Nagelkerke’s pseudo-R^2^ was calculated to quantify explanatory power. To address potential sparse-data bias and model instability due to the limited number of events, we performed sensitivity analyses using Firth penalized logistic regression (37).

#### 2.3.2 Internal validation and robustness assessment

Bootstrap validation: Internal validation was performed using bootstrap resampling (2000 iterations) to obtain optimism-corrected estimates of model performance.

Ridge regression: This penalized regression was applied to stabilize coefficient estimates and mitigate overfitting.

Random forest benchmarking: A random forest model (2000 trees) was implemented as an exploratory approach to evaluate whether nonlinear relationships among candidate variables could provide additional discriminative information beyond the prespecified logistic regression framework (38).

Model performance: Discrimination was evaluated using the AUC estimates obtained under the corresponding validation procedures.

#### 2.3.3 Secondary analyses

Exploratory analyses assessed associations between preoperative phase angle (PhA_Pre) and secondary postoperative outcomes, including overall complications (Complications), infectious complications (Infec_complications), and length of hospital stay (LOS_Post). Logistic regression was applied for binary outcomes (overall and infectious complications), and negative binomial regression was used for the count outcome (LOS_Post). To maintain analytical consistency with the primary analysis, all multivariate models were adjusted for the same pre-specified covariates: gender, age, NRS2002 (dichotomized as ≥ 3 or < 3), PALB, and baseline sarcopenia status (Sarcopenia_Pre).

## 3 Results

### 3.1 Patient characteristics and clinical outcomes

**FIGURE 1:**
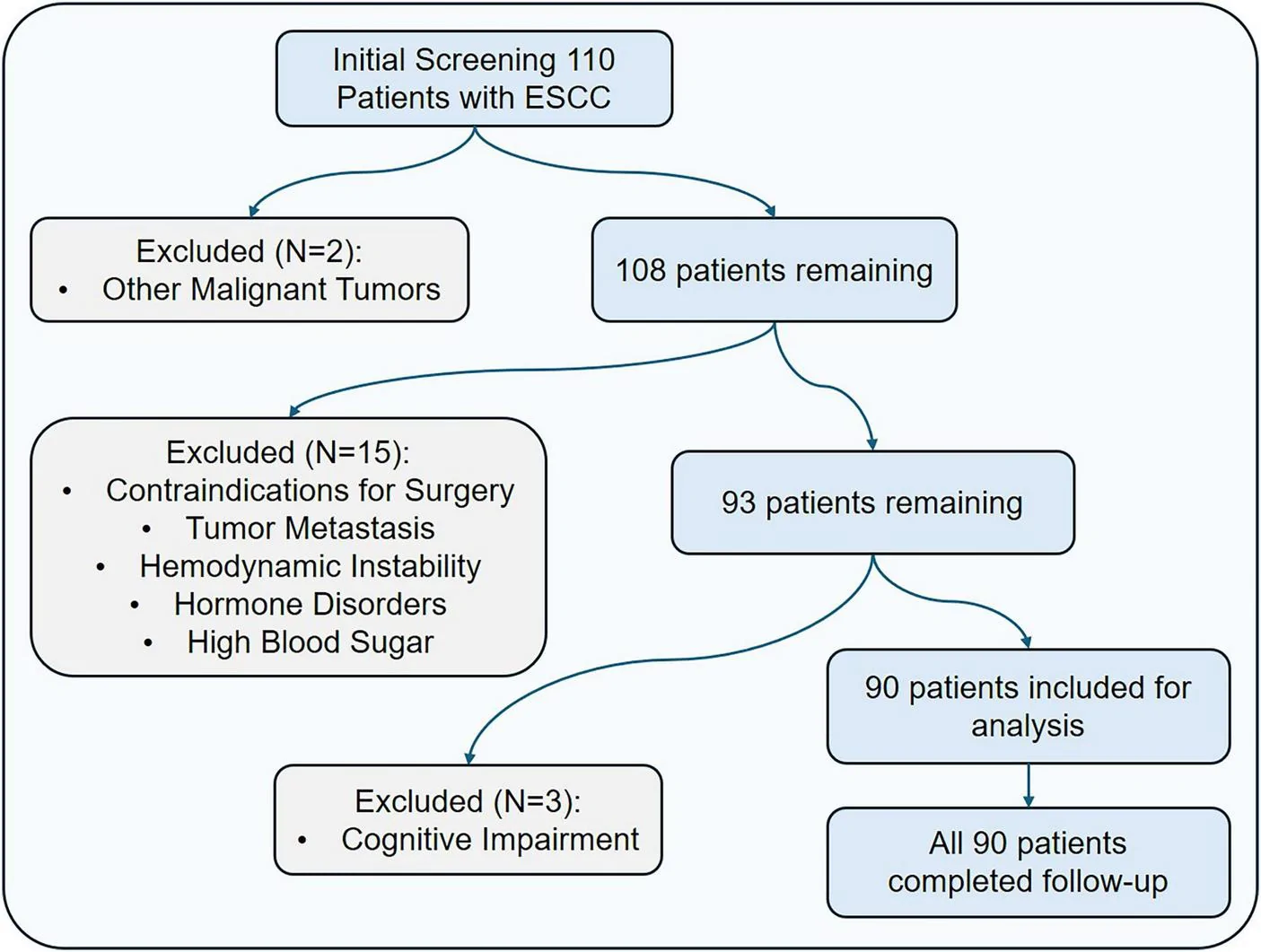
Flow chart for the selection of included subjects. ESCC, esophageal squamous cell carcinoma.

From an initial recruitment of 110 ESCC patients, 20 were excluded due to other malignancies, surgical contraindications, or cognitive impairment. The final cohort comprised 90 patients who completed postoperative follow-up (Figure 1).

Baseline demographics, clinical characteristics and postoperative outcomes, including sarcopenia, overall complications, infectious complications, and length of hospital stay, are summarized in Table 1. Pulmonary infections were the most common complication, followed by incision infections and anastomotic leaks or stenosis. Less frequent complications included arrhythmias, intestinal obstruction, pneumothorax, and pressure injuries. Nutritional and biochemical status before and after surgery are presented in Table 2, highlighting significant perioperative declines in phase angle and body weight, along with increases in nutritional risk and malnutrition prevalence.

**TABLE 1:** Baseline characteristics and clinical outcomes of included patients (*N* = 90).

Variable	Value
Baseline characteristics	
Gender (Female)	17 (18.9%)
Age (years)	61.13 ± 7.92
40–50	11 (12.2%)
51–60	32 (35.6%)
61–70	36 (40.0%)
71–80	11 (12.2%)
BMI	23.12 ± 3.34
Underweight	3 (3.3%)
Normal weight	55 (61.1%)
Pre-obesity	24 (26.7%)
Obesity	8 (8.9%)
Cancer stage	
I	18 (20.0%)
II	38 (42.2%)
III	32 (35.6%)
IV	2 (2.2%)
Comorbidities (No)	58 (64.4%)
Hypertension (No)	60 (66.7%)
Diabetes mellitus (No)	81 (90.0%)
Cardiovascular diseases (No)	84 (93.3%)
History of smoking (No)	28 (31.1%)
History of drinking alcohol (No)	37 (41.1%)
Preoperative chemotherapy (No)	52 (57.8%)
Use of protein powder (No)	47 (52.2%)
Use of enteral nutrition preparation (No)	58 (77.8%)
Clinical outcomes	
Sarcopenia_Post	17 (18.9%)
Complications (Yes)	37 (41.1%)
Pulmonary infection	17 (18.9%)
Incisional infection	13 (14.4%)
Anastomotic leakage	9 (10%)
Anastomotic stenosis	3 (3.3%)
Arrhythmia	3 (3.3%)
Intestinal obstruction	1 (1.1%)
Infectious complications (Yes)	28 (31.1%)
LOS_Post (days)	15.0 (13.0–20.0)

Data are shown as total number plus percentage (in parenthesis) among all participants [*n* (%)] or mean ± standard deviation or median (interquartile range), as appropriate. Weight class was set according to the BMI (body mass index) definitions for adults by the Chinese guidelines (63). LOS_Post, postoperative length of hospital stay.

**TABLE 2:** Changes in nutritional status related variables from pre- to postoperative period (*N* = 90).

Variable	Pre-operation	Post-operation	*p* value	Effect size
Sarcopenia (Yes)	11 (12.2%)	17 (18.9%)	0.149^a^	3.00^d^
NRS2002 (≥3)	13 (14.4%)	42 (46.7%)	<0.001^a^	5.14^d^
PG-SGA (≥4)	21 (23.3%)	47 (52.2%)	<0.001^a^	3.60^d^
Grip strength (kg)	30.75 (24.43–36.77)	28.50 (23.05–32.80)	<0.001^b^	–0.55^e^
Energy intake (kcal/day)	1770.28 ± 361.14	1996.61 ± 376.13	<0.001^c^	0.43^f^
Protein intake (g/day)	76.31 ± 21.20	95.42 ± 20.75	<0.001^c^	0.66^f^
PhA (°)	5.16 ± 0.70	4.84 ± 0.69	<0.001^c^	–0.96^f^
Body weight (kg)	65.36 ± 10.20	63.11 ± 9.75	<0.001^c^	–1.00^f^
BMI (kg/m^2^)	23.12 ± 3.34	22.34 ± 3.36	<0.001^c^	–1.00^f^
TBW (kg)	36.70 ± 5.44	35.43 ± 5.14	<0.001^c^	–0.94^f^
ECW (kg)	14.17 ± 2.04	13.78 ± 1.96	<0.001^c^	–0.67^f^
ICW (kg)	22.53 ± 3.42	21.65 ± 3.20	<0.001^c^	–1.08^f^
ECW/TBW (%)	38.64 ± 0.74	38.92 ± 0.69	<0.001^c^	0.59^f^
PBF (%)	23.46 ± 7.59	23.32 ± 7.54	0.468^c^	–0.08^f^
BMR (kcal/d)	1444.72 ± 159.16	1409.06 ± 150.49	<0.001^c^	–0.90^f^
BCM (kg)	32.27 ± 4.91	31.00 ± 4.59	<0.001^c^	–1.10^f^
AMC (cm)	26.85 ± 2.30	26.33 ± 2.21	<0.001^c^	–0.79^f^
FFMI (kg/m^2^)	17.53 ± 1.81	16.95 ± 1.76	<0.001^c^	–0.91^f^
ALB (g/L)	43.96 ± 3.28	41.42 ± 3.84	<0.001^c^	–0.67^f^
PALB (g/L)	0.25 (0.23–0.29)	0.25 (0.18–0.27)	0.018^b^	–0.29^e^
Crea (μmol/L)	66.51 ± 12.33	58.91 ± 12.55	<0.001^c^	–0.81^f^
WBC (10^9^/L)	6.60 ± 2.24	6.90 ± 2.08	0.348^c^	0.10^f^
HGB (g/L)	138.46 ± 15.47	121.46 ± 15.09	<0.001^c^	–1.24^f^

Data are presented as mean ± standard deviation, median (interquartile range), or *n* (%), as appropriate.

^a^McNemar’s test;

^b^Wilcoxon signed-rank test;

^c^paired *t*-test.

^d^odds ratio;

^e^rank-biserial correlation;

^f^Cohen’s d. AMC, arm muscle circumference; ALB, albumin; BCM, body cell mass; BMR, basal metabolic rate; BMI, body mass index; Crea, serum creatinine; ECW, extracellular water; ECW/TBW (%), percentage of extracellular water to total body water ratio; FFMI, fat-free mass index; HGB, hemoglobin; ICW, intracellular water; NRS2002, Nutritional Risk Screening 2002; PhA, phase angle; PALB, prealbumin; PBF%, percentage body fat; PG-SGA, patient-generated subjective global assessment; TBW, total body water; WBC, white blood cell.

### 3.2 Association between perioperative phase angle decline and postoperative sarcopenia

#### 3.2.1 Stratified analyses

Patients were dichotomized according to the mean perioperative phase angle decline (ΔPhA = 0.32°) into Low ΔPhA (<0.32°) and High ΔPhA (≥0.32°) groups and compared clinical outcomes between both groups (Table 3).

**TABLE 3:** Comparison of clinical outcomes according to ΔPhA_Cat groups (*N* = 90).

Variable	Low Δ PhA (*n* = 53)	High Δ PhA (*n* = 37)	*p* value
Sarcopenia_Post			0.055^a^
No	47 (88.7%)	26 (70.3%)	
Yes	6 (11.3%)	11 (29.7%)	
Complications			0.319^a^
No	34 (64.2%)	19 (51.4%)	
Yes	19 (36.8%)	18 (48.6%)	
Infectious complications			0.357^a^
No	39 (73.6%)	23 (62.2%)	
e Yes	14 (26.4%)	14 (37.8%)	
LOS_Post (days)	15 (13–19)	15 (14–21)	0.341^b^

Data are presented as *n* (%) or median (interquartile range), as appropriate.

^a^Pearson’s χ^2^ test;

^b^Wilcoxon rank sum test. Low ΔPhA, phase angle decline <0.32°; High ΔPhA, phase angle decline ≥0.32°; LOS_Post, postoperative length of hospital stay.

The incidence of postoperative sarcopenia (Sarcopenia_Post) tended to be higher in the High ΔPhA group than in the Low ΔPhA group (29.7% vs. 11.3%, *p* = 0.055), trending toward significance. Rates of overall complications (48.6% vs. 36.8%, *p* = 0.319) and infectious complications (37.8% vs. 26.4%, *p* = 0.357) were higher in the High ΔPhA group, but the differences were not statistically significant. Median postoperative length of hospital stay was similar between groups [15 (14–21) vs. 15 (13–19) days, *p* = 0.341].

#### 3.2.2 Univariate analyses and pairwise correlation check

Results of univariate logistic regression are presented as odds ratios (ORs) with 95% confidence intervals (CIs) and *p* values (Supplementary Table 1). Several variables were associated with postoperative sarcopenia at the *p* < 0.10 level, with odds ratios distinct from 1, including ΔPhA_Cat_Mean, NRS2002_Cat, Sarcopenia_Pre, BMI, AMC and ECW/TBW (%). Pairwise correlation analysis identified several variable pairs with high collinearity (| ρ| > 0.7) in Supplementary Table 2, such as BMI with AMC, AMC with TBW and TBW with ECW.

#### 3.2.3 Multivariable analysis and model performance

The association between perioperative phase angle decline (ΔPhA_Cat) and postoperative sarcopenia was evaluated using multivariable logistic regression. The model was adjusted for preoperative sarcopenia status (Sarcopenia_Pre) and nutritional risk (NRS2002_Cat, ≥ 3). BMI, AMC and ECW/TBW (%), which were significant in univariate analysis, were not retained in the multivariable model following validation analyses (Supplementary Table 3). Adjusted ORs with 95% CIs are presented in Table 4. In sensitivity analyses treating ΔPhA as a continuous variable, each 0.1° greater perioperative decline in PhA was associated with higher odds of postoperative sarcopenia, while the association did not reach statistical significance (OR = 1.09, 95% CI: 0.90–1.32, *p* = 0.387; Supplementary Table 4).

**TABLE 4:** Multivariable logistic regression for postoperative sarcopenia (*N* = 90).

Variable	Primary model aOR (95% CI)	*p* value	Extended model aOR (95% CI)	*p* value
ΔPhA_Cat (High vs. Low)	4.69 (1.08–20.45)	0.040	4.71 (1.08–21.97)	0.045
NRS2002_Cat (≥3 vs. <3)	8.57 (1.73–42.49)	0.008	7.83 (1.24–49.50)	0.029
Sarcopenia_Pre (Yes vs. No)	21.59 (3.49–133.96)	<0.001	24.75 (3.59–170.10)	0.001
Gender (Male vs. Female)	–	–	1.07 (0.16–7.30)	0.944
Age (years)	–	–	1.03 (0.94–1.14)	0.507
PALB (g/L)	–	–	6.98 (0.00–1.80 × 10^10^)	0.857

Low ΔPhA, phase angle decline <0.32°; High ΔPhA, phase angle decline ≥0.32°; NRS2002_Cat, dichotomized NRS2002 as ≥3 or <3; Sarcopenia_Pre, preoperative sarcopenia; PALB, prealbumin.

**FIGURE 2:**
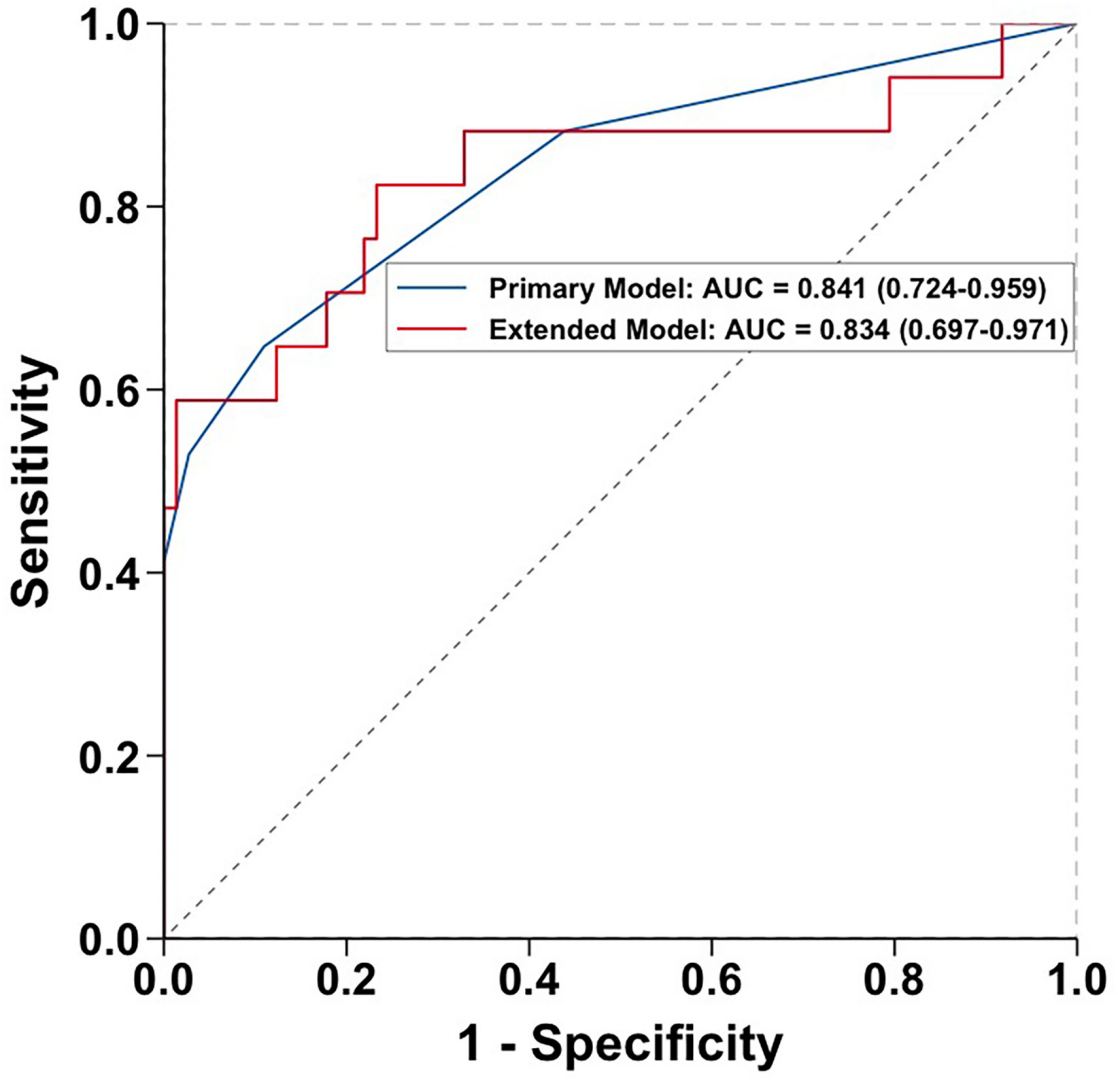
ROC curves illustrating discriminative performance of multivariable models for postoperative sarcopenia.

The primary model demonstrated strong discriminatory power for postoperative sarcopenia, with an area under the receiver operating characteristic (ROC) curve of 0.841 (95% CI: 0.724–0.959) (Figure 2). The extended model showed comparable performance (AUC = 0.834, 95% CI: 0.697–0.971), without significant improvement after adding gender, age, and PALB (DeLong’s test, *p* = 0.677). Nagelkerke’s pseudo-R^2^ values of 0.469 for the primary model and 0.474 for the extended model further supported the strong explanatory power of both models.

#### 3.2.4 Model robustness and validation

**FIGURE 3:**
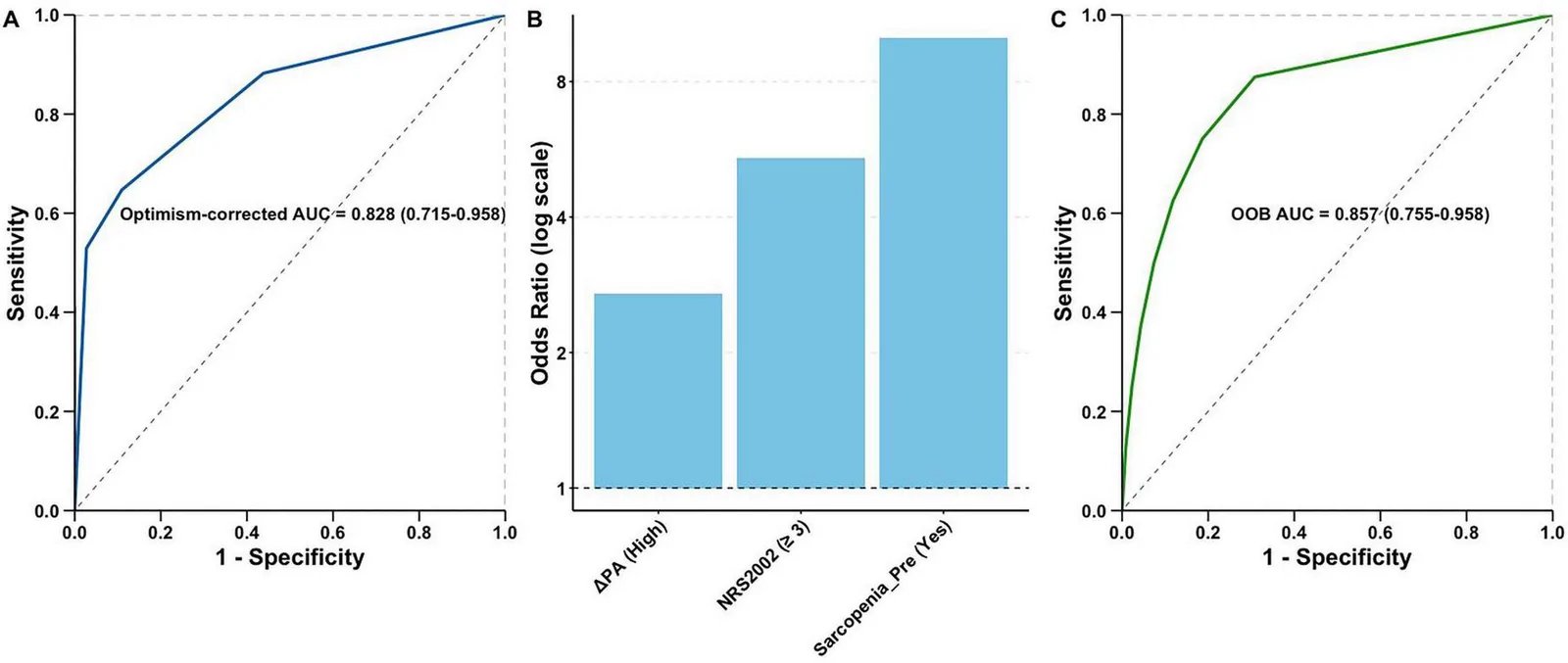
Validation of the association between perioperative phase angle decline and postoperative sarcopenia using internal validation methods. **(A)** Optimism-corrected AUC from 2,000 bootstrap resamples. **(B)** Ridge regression showing adjusted odds ratios (ORs) for model covariates. The ridge regression was performed under two penalization thresholds: (min optimal penalty, minimizing cross-validation error) and (1se more conservative penalty, within one standard error of the minimum). The (1se threshold applies stronger shrinkage, penalizing less stable variables more aggressively. The preservation of (PhA_Cat, Sarcopenia_Pre, and NRS2002_Cat coefficients under both thresholds confirms their stability and independent contribution. **(C)** Random forest variable importance plot (2,000 trees). OOB AUC, out-of-bag AUC (64).

The stability and robustness of the final model were assessed using three complementary approaches. First, bootstrap validation (2,000 replicates) yielded an optimism-corrected AUC of 0.828 (95% CI: 0.715–0.958), closely aligned with the naïve AUC of 0.841 (95% CI: 0.724–0.959), indicating minimal overfitting (Figure 3A). Second, ridge regression confirmed coefficient stability, with ΔPhA_Cat, preoperative sarcopenia, and NRS2002 ≥ 3 consistently retained as independent correlates (Figure 3B). Besides, random forest benchmarking (2,000 trees) produced an out-of-bag AUC of 0.857 (95% CI: 0.755–0.958), comparable to logistic regression, indicating that machine-learning benchmark model offered no meaningful gain in discrimination (Figure 3C). Sensitivity analyses using Firth penalized logistic regression yielded a similar association between ΔPhA_Cat and postoperative sarcopenia, supporting the consistency of the primary finding. The adjusted odds ratio for ΔPhA_Cat remained significant (aOR = 3.95, 95% CI: 1.08–17.59, *p* = 0.037), with estimates slightly attenuated under penalized regression (Supplementary Table 5).

### 3.3 Secondary analyses

Secondary analyses assessed the association between preoperative phase angle and other postoperative outcomes. After adjustment for the same covariates as the extended model of the primary analyses (age, gender, NRS2002_Cat, PALB, Sarcopenia_Pre), preoperative phase angle was not significantly associated with the risk of postoperative overall complications (OR = 2.05, 95% CI: 0.85–4.95, *p* = 0.111), infectious complications (OR = 2.21, 95% CI: 0.85–5.73, *p* = 0.104), or length of hospital stay (IRR = 1.18, 95% CI: 0.995–1.39, *p* = 0.056).

## 4 Discussion

This study, focusing on esophageal squamous cell carcinoma (ESCC) patients, provided an innovative and comprehensive analysis on the correlations between postoperative sarcopenia and perioperative phase angle decline. Dynamic phase angle decline around the perioperative period (ΔPhA) was heightened, and using the overall mean of ΔPhA among patients as a cut-off was initially proposed and deeply analyzed. To our knowledge, this is the first study which investigated the correlations between postoperative sarcopenia and perioperative phase angle decline rather than the baseline value. Given that China carries a disproportionate burden of ESCC, accounting for more than 40% of all new cases and deaths worldwide under the context of consistently increasing global ESCC burden (1, 39), these population-specific data are crucial for guiding clinical management. Moreover, studies specifically addressing PhA in ESCC surgical cohorts remain scarce. Our findings suggest that ΔPhA provides valuable discriminative ability in Chinese ESCC inpatients, with potential relevance both locally and globally for timely perioperative intervention and improvement of long-term outcomes.

To place our findings in a broader clinical context, the overall complication rate in our cohort was 41.1%, which is comparable to rates reported in contemporary esophagectomy cohorts, including 59% in a large international prospective study of 2,704 esophagectomies (13) and 50.9% in a recent cohort of 450 patients undergoing curative esophagectomy (40). Differences in patient characteristics, surgical approaches, perioperative management, and complication definitions should be considered when interpreting these comparisons. Postoperative sarcopenia occurred in 18.9% of our cohort; however, direct comparison with previous studies is limited by differences in diagnostic criteria and assessment timing. Given the concurrent timing of ΔPhA and postoperative sarcopenia assessments, our findings indicate an association rather than prediction.

Phase angle (PhA) is a composite measure derived from bioelectrical impedance analysis (BIA), reflecting cell membrane integrity and body composition (15). However, its reliability as a standalone marker in cancer patients could be compromised by factors such as inflammation, fluid balance, and tumor stage (41, 42). Specifically, PhA may underestimate true muscle mass in the presence of edema and overestimate it during dehydration (42, 43). Consequently, monitoring dynamic changes in PhA over time is recommended to obtain a more accurate assessment of the longitudinal physiological impact of surgical stress and catabolism, offering a potentially superior biomarker for postoperative sarcopenia. Although ΔPhA was approximately normally distributed, we categorized it into a binary variable (ΔPhA_Cat: high vs. low decrease) using its mean value ΔPhA = 0.32° as a cutoff to generate a clinically usable threshold. Given the difficulty of preventing postoperative decline, such a threshold offers a pragmatic clinical approach to identify patients at higher risk of sarcopenia.

Variables were first explored in univariate analyses, then stratified analyses, and finally entered into multivariate models, ensuring consistency and avoiding spurious associations. In this prospective study of 90 ESCC patients undergoing esophagectomy, we observed that a higher level of perioperative decline in phase angle (ΔPhA ≥ 0.32°) was associated with postoperative sarcopenia, independent of preoperative sarcopenia and nutritional risk. In the multivariable model, preoperative sarcopenia and NRS2002 ≥ 3 showed larger effect sizes than ΔPhA, but ΔPhA remained independently significant, suggesting that baseline disease status and nutritional vulnerability represent stronger preoperative determinants, whereas ΔPhA reflects an additional and distinct perioperative physiological response. Conversely, preoperative PhA alone was not significantly associated with postoperative sarcopenia, underscoring the importance of dynamic assessment. While a cross-sectional study in gastric cancer patients showed that lower preoperative PhA was associated with preoperative sarcopenia (44), our longitudinal data suggest that perioperative PhA decline provides additional information beyond static baseline assessment.

Both a primary and an extended model were evaluated. The primary model, which included ΔPhA_Cat, preoperative sarcopenia, and nutritional risk (NRS2002_Cat), was selected as the main analytic framework because it provided a set of correlates that was parsimonious, clinically interpretable, and statistically stable. The extended model, which additionally incorporated age, gender, and PALB, did not materially improve model discrimination or calibration compared with the primary model. Given the relatively small number of outcome events, the additional covariates may have provided limited incremental information while increasing model complexity and the risk of instability.

Internal validation methods provide powerful tools for the internal validation of linear regression and clinical prediction models (45, 46). After employing ridge regression to account for potential multicollinearity, the odds ratio for perioperative ΔPhA, along with those for baseline sarcopenia and NRS2002, remained statistically significant and clinically meaningful. Moreover, both the optimism-corrected AUC obtained via bootstrap validation and the out-of-bag (OOB) AUC from random forest benchmarking support the robustness and reliability of ΔPhA as a discriminative marker for postoperative sarcopenia.

Our findings affirm the established role of PhA as a critical marker of cellular health and nutritional status in oncology populations (47–49). Prior studies have demonstrated that low PhA is associated with postoperative morbidity (23), impaired recovery (50), and malnutrition or sarcopenia (51) in esophageal cancer. The novelty of this study lies in evaluating perioperative PhA decline, rather than preoperative PhA alone, in relation to postoperative sarcopenia. This aligns with the pathophysiological understanding that surgical stress triggers catabolism and muscle loss, which may not be adequately captured by static preoperative measures (52).

Traditional markers such as BMI, weight, or single-timepoint body composition assessments may overlook these subtle yet clinically relevant changes (52, 53). This was evident in our analysis, where BMI exhibited an apparent protective association in univariate analysis but demonstrated substantial instability under rigorous validation. Ridge regression, a technique designed to reduce overfitting by penalizing less important variables, substantially shrank the coefficient for BMI toward the null (from –0.16 at the optimal fit to –0.06 under stronger regularization), while preserving meaningful coefficients for phase angle decline and baseline sarcopenia. This may partly reflect its correlation with preoperative sarcopenia (Spearman ρ = –0.45, *p* < 0.001) and other confounders, suggesting that the apparent protective effect of BMI in univariate analysis is consistent with the well-documented limitations of BMI as a measure of body composition in cancer patients, where fluid shifts and sarcopenic obesity can confound interpretation (54, 55). Similarly, AMC and ECW/TBW (%) were excluded due to high bootstrap coefficient of variation (>150%) and ridge regression coefficients shrunk close to zero. Collectively, these findings reinforce the potential of ΔPhA as a robust and biologically plausible marker.

Our findings offer several clinical implications. First, ΔPhA may help characterize patients with postoperative muscle deterioration and may warrant closer nutritional evaluation and rehabilitative interventions such as enhanced protein supplementation (56), tailored exercise programs (57), or closer postoperative monitoring (58), to mitigate or even reverse the phase angle decline. Second, the use of dynamic PhA assessment rather than static preoperative measures could optimize risk stratification and resource allocation in the perioperative period. Finally, we present a methodological framework demonstrating how ridge regression, bootstrap validation and random forest can internally validate model stability and internal optimism, ensuring the findings are robust and clinically interpretable.

Exploratory analyses found no significant association between preoperative PhA and overall or infectious complications, or length of stay (LOS). Although preoperative PhA is a useful baseline nutritional marker, its limited predictive value for complications or LOS post surgery in our cohort suggests that dynamic measures may be more relevant for perioperative risk assessment. While the point estimates suggested possible associations (OR = 2.05 for overall complications and OR = 2.21 for infectious complications), the wide confidence intervals indicate substantial uncertainty. The limited number of events may have reduced the power to detect modest associations in these exploratory analyses.

These results indicate that ΔPhA may be particularly useful for postoperative sarcopenia stratification, while its associations with other outcomes may require a larger sample size for detection and is likely influenced by other factors, including surgical technique, comorbidities, and perioperative management.

Our findings must be considered in the context of the evolving literature on body composition and surgical outcomes. A scoping review of preoperative risk factors for postoperative physical frailty in esophageal cancer identified limited evidence on factors associated with postoperative functional decline, with existing studies largely focusing on preoperative characteristics (59). Our study provides preliminary evidence that perioperative changes in PhA may be associated with postoperative sarcopenia. These findings complement observations in gastric cancer surgery, where sarcopenia showed prognostic relevance but inconsistent associations with postoperative complications (20), suggesting that the prognostic implications of sarcopenia may differ by cancer type and warrant cancer-specific investigations.

Several methodological strengths enhance the validity of our findings. First, the prospective, longitudinal design with standardized phase angle measurements and comprehensive nutritional assessments provides high-quality data. Second, we employed multiple complementary validation methods, including bootstrap validation, ridge regression, and random forest benchmarking, ensuring model stability and minimizing overfitting. Besides, the study used clinically relevant covariates (baseline sarcopenia, NRS2002, PALB, age, gender) selected *a priori*, enhancing interpretability and avoiding data-driven bias. Importantly, we also demonstrate the value of bootstrap validation and penalized ridge regression analysis in identifying unstable variable candidates, such as BMI, AMC and ECW/TBW (%), which may appear statistically significant in conventional models but lack stability and reliable effect size. This approach strengthens confidence in the clinical relevance of the final model and reduces the risk of misleading interpretations.

Nevertheless, limitations must be acknowledged. First, the modest sample size (*N* = 90, with only 17 postoperative sarcopenia events, including 9 new cases) limited statistical power and the precision of the estimated associations, as reflected by the relatively wide confidence intervals. The AUC estimate also had a relatively wide confidence interval (0.841, 95% CI: 0.724–0.959), indicating uncertainty in model discrimination. Therefore, the findings should be considered exploratory and hypothesis-generating and require validation in larger, adequately powered cohorts. However, sensitivity analyses using Firth penalized logistic regression yielded similar results, supporting the robustness of the primary findings. PALB was explored as a covariate in the extended model but exhibited coefficient instability, likely due to sparse-data bias. Therefore, findings from the extended model should be interpreted with caution. Second, the postoperative phase angle and postoperative sarcopenia were assessed at the same postoperative time point (1 month after surgery). While this does not diminish the clinical relevance of the observed association, it precludes claims of predictive utility (60, 61). Third, the study cohort consisted exclusively of ESCC patients treated at a single tertiary cancer center. Although the relatively homogeneous population and standardized care setting may reduce heterogeneity within the cohort, they also limit the generalizability of our findings to other populations, including patients with esophageal adenocarcinoma, those treated in non-tertiary centers, or those receiving different surgical approaches. External validation in diverse, multi-center cohorts is essential before clinical translation. Fourth, the mean value of ΔPhA (0.32°) was selected as an exploratory data-driven threshold for clinical interpretability rather than a biologically established cutoff. When ΔPhA was modeled as a continuous variable, no significant linear association was observed, raising the possibility that the relationship may not be adequately described by a simple linear model. Further external validation in independent cohorts is required to establish a clinically generalizable threshold. Finally, although exploratory analyses adjusted for key covariates, the influence of unmeasured confounding cannot be excluded.

Future studies are needed to validate and translate our findings into clinical practice. The proposed threshold for a clinically meaningful decline in phase angle (ΔPhA = 0.32°) should be confirmed in larger, multi-center cohorts. Additionally, future studies incorporating earlier postoperative PhA measurements (e.g., 1–2 weeks post-surgery) are needed to determine whether ΔPhA can serve as an early predictive indicator of postoperative sarcopenia development. Ultimately, interventional studies are required to determine whether mitigating PhA decline through nutritional or rehabilitative strategies can improve recovery and reduce sarcopenia incidence (62).

Although postoperative sarcopenia can be directly assessed, serial phase angle measurement may provide complementary information regarding dynamic changes in cellular integrity and nutritional status during recovery. Compared with comprehensive sarcopenia assessment (which requires muscle mass measurement, grip strength, and gait speed), bioelectrical impedance-derived phase angle can be obtained rapidly and repeatedly in routine clinical practice, making it a potentially useful monitoring parameter. The association between perioperative phase angle decline and sarcopenia identified in this study suggests that phase angle trajectory may serve as a practical surrogate marker for muscle health during the postoperative period.

## 5 Conclusions

Perioperative decline in phase angle (ΔPhA) demonstrates a clinically meaningful association with postoperative sarcopenia in patients undergoing esophagectomy for ESCC, whereas preoperative PhA alone was not significantly associated with postoperative sarcopenia or postoperative complications. Given the concurrent assessment of postoperative PhA and sarcopenia, these findings indicate association rather than prediction. Incorporating serial PhA measurements into perioperative assessment may provide complementary information on postoperative muscle health and help characterize patients who may warrant closer nutritional and functional assessment. The cohort-derived ΔPhA threshold of 0.32° should be considered exploratory and requires validation in larger, multicenter cohorts. Future studies should evaluate earlier postoperative PhA trajectories and determine whether dynamic PhA changes provide clinically useful information beyond established measures of muscle mass, muscle strength, and nutritional status, and whether such information can ultimately support individualized nutritional and rehabilitative strategies.

## Data Availability

The original contributions presented in the study are included in the article/Supplementary material, further inquiries can be directed to the corresponding authors.
