# Supplementary material for: Perioperative phase angle decline is associated with postoperative sarcopenia in esophageal cancer: a longitudinal cohort study with internal validation
Source: Front Nutr. 2026 Sep 4;13:1926195. doi: 10.3389/fnut.2026.1926195 (PMC13585515; doi:10.3389/fnut.2026.1926195)
Supplement: Supplementary file 1 [file Table_1.docx]

Supplementary Material

# 1 Supplementary Figures and Tables

## **TABLE S1.** Univariate logistic regression analyses of factors associated with postoperative sarcopenia (N = 90).

| **Variable** | **OR** | **95% CI** | ***p* value** |
| --- | --- | --- | --- |
| Gender (Male vs. Female) | 0.7 | 0.21–2.81 | 0.589 |
| Age (years) | 1.04 | 0.98–1.12 | 0.226 |
| Weight (kg) | 0.76 | 0.65–0.86 | < 0.001 |
| BMI (kg/m²) | 0.48 | 0.31–0.67 | < 0.001 |
| Tumor stage (III–IV vs. I–II) | 1.61 | 0.54–4.70 | 0.383 |
| Comorbidities (Yes vs. No) | 0.71 | 0.21–2.14 | 0.558 |
| Smoking (Yes vs. No) | 0.79 | 0.27–2.54 | 0.68 |
| Alcohol (Yes vs. No) | 0.74 | 0.25–2.19 | 0.581 |
| Preoperative chemotherapy (Yes) | 0.51 | 0.15–1.51 | 0.24 |
| Protein powder use (Yes vs. No) | 0.72 | 0.24–2.08 | 0.546 |
| Enteral nutrition (Yes vs. No) | 2.45 | 0.83–7.32 | 0.102 |
| Sarcopenia_Pre (Yes vs. No) | 20.74 | 5.05–109.17 | < 0.001 |
| NRS2002_Cat (≥ 3 vs. <3) | 12.09 | 3.35–48.45 | < 0.001 |
| PG-SGA_Cat (> 3 vs. ≤ 3) | 2.95 | 0.93–9.13 | 0.06 |
| Grip strength (kg) | 0.9 | 0.83–0.96 | 0.005 |
| Energy intake (kcal/d) | 1 | 1.00–1.00 | 0.508 |
| Protein intake (g/d) | 0.97 | 0.95–1.00 | 0.062 |
| Preoperative PhA (°) | 0.46 | 0.20–1.02 | 0.065 |
| ΔPhA_Cat (High vs. Low) | 3.31 | 1.13–10.61 | 0.033 |
| TBW (L) | 0.82 | 0.72–0.91 | 0.001 |
| ECW (L) | 0.59 | 0.42–0.80 | 0.001 |
| ICW (L) | 0.72 | 0.59–0.86 | 0.001 |
| ECW/TBW (%) | 2.14 | 1.01–4.93 | 0.057 |
| PBF (%) | 0.91 | 0.83–0.98 | 0.02 |
| BMR (kcal/d) | 0.99 | 0.99–1.00 | 0.001 |
| AMC (cm) | 0.47 | 0.30–0.66 | < 0.001 |
| PALB (g/L) | 0 | 0.00–86.24 | 0.205 |
| Creatinine (µmol/L) | 0.96 | 0.92–1.01 | 0.136 |
| WBC (×109/L) | 0.89 | 0.65–1.14 | 0.4 |
| HGB (g/L) | 0.98 | 0.94–1.01 | 0.226 |

Note: Low ΔPhA, phase angle decline < 0.32°; High ΔPhA, phase angle decline ≥ 0.32°; AMC, arm muscle circumference; ALB, albumin; BCM, body cell mass; BMR, basal metabolic rate; BMI, body mass index; Crea, serum creatinine; ECW, extracellular water; ECW/TBW (%), percentage of extracellular water to total body water ratio; FFMI, fat-free mass index; HGB, haemoglobin; ICW, intracellular water; NRS2002, Nutritional Risk Screening 2002; PhA, phase angle; PALB, prealbumin; PBF%, percentage body fat; PG-SGA, patient-generated subjective global assessment; TBW, total body water; WBC, white blood cell.

## **TABLE S2.** Results of pairwise correlation analysis.

| **Variable** | **Gender** | **Age** | **Weight** | **BMI** | **Stage_Cat** | **Comorbidities_Pre** | **Smoking** | **Alcohol** | **Pre_chemotherapy** | **Protein_powder** | **ENP** | **Sarcopenia_Pre** | **NRS2002_Cat** | **PG_SGA_Cat2** | **Grip_strength** | **Energy** | **Protein** | **PhA** | **Delta_PhA_Cat_Mean** | **TBW** | **ECW** | **ICW** | **ECW_TBW_percent** | **PBF** | **BMR** | **AMC** | **PALB** | **Crea** | **WBC** | **HGB** |
| --- | --- | --- | --- | --- | --- | --- | --- | --- | --- | --- | --- | --- | --- | --- | --- | --- | --- | --- | --- | --- | --- | --- | --- | --- | --- | --- | --- | --- | --- | --- |
| Gender | 1 | -0.4** | 0.31* | -0.04 | 0.02 | -0.06 | 0.6** | 0.52** | 0.07 | 0.18 | 0 | -0.08 | -0.12 | -0.2 | 0.66** | 0.47** | 0.39** | 0.56** | 0 | 0.65** | 0.64** | 0.66** | -0.46** | -0.57** | 0.65** | 0.38** | 0.41** | 0.34* | 0.09 | 0.37** |
| Age | -0.4** | 1 | -0.19 | 0 | -0.06 | 0.18 | -0.22* | -0.25* | -0.01 | -0.03 | 0.16 | 0.04 | 0.28* | 0.21* | -0.44** | -0.22* | -0.25* | -0.5** | -0.08 | -0.3* | -0.26* | -0.33* | 0.54** | 0.23* | -0.31* | -0.25* | -0.28* | -0.01 | -0.08 | -0.25* |
| Weight | 0.31* | -0.19 | 1 | 0.84** | -0.07 | 0.1 | 0.11 | 0.19 | 0.09 | 0.09 | -0.18 | -0.49** | -0.34* | -0.22* | 0.5** | 0.2 | 0.25* | 0.34* | -0.11 | 0.8** | 0.78** | 0.8** | -0.24* | 0.35** | 0.8** | 0.9** | 0.2 | 0.18 | 0.01 | 0.16 |
| BMI | -0.04 | 0 | 0.84** | 1 | -0.06 | 0.14 | -0.08 | 0.01 | 0.01 | -0.02 | -0.22* | -0.45** | -0.31* | -0.07 | 0.2 | 0.03 | 0.11 | 0.31* | -0.11 | 0.46** | 0.43** | 0.47** | -0.19 | 0.63** | 0.46** | 0.83** | 0.12 | 0.15 | 0.03 | 0.07 |
| Stage_Cat | 0.02 | -0.06 | -0.07 | -0.06 | 1 | 0.04 | -0.12 | 0.09 | -0.06 | 0.03 | 0.09 | -0.01 | 0.14 | 0.11 | 0.05 | -0.13 | -0.01 | 0.04 | 0 | -0.06 | -0.07 | -0.05 | -0.01 | 0.01 | -0.06 | -0.03 | 0.05 | -0.03 | -0.01 | 0.11 |
| Comorbidities_Pre | -0.06 | 0.18 | 0.1 | 0.14 | 0.04 | 1 | -0.15 | 0.15 | 0.07 | 0.08 | 0.13 | -0.21 | -0.04 | -0.08 | -0.13 | -0.14 | -0.07 | -0.18 | -0.05 | 0.05 | 0.07 | 0.03 | 0.17 | 0.13 | 0.04 | 0.03 | -0.02 | -0.04 | 0.12 | -0.22* |
| Smoking | 0.6** | -0.22* | 0.11 | -0.08 | -0.12 | -0.15 | 1 | 0.51** | -0.11 | 0.02 | -0.05 | -0.04 | 0 | -0.08 | 0.32* | 0.44** | 0.31* | 0.34* | -0.07 | 0.28* | 0.27* | 0.29* | -0.34* | -0.32* | 0.29* | 0.11 | 0.13 | 0.03 | 0.27* | 0.28* |
| Alcohol | 0.52** | -0.25* | 0.19 | 0.01 | 0.09 | 0.15 | 0.51** | 1 | -0.15 | 0.03 | -0.04 | -0.17 | 0.02 | -0.18 | 0.39** | 0.31* | 0.2 | 0.45** | -0.13 | 0.37** | 0.34* | 0.38** | -0.43** | -0.33* | 0.37** | 0.25* | 0.27* | 0.14 | 0.3* | 0.29* |
| Pre_chemotherapy | 0.07 | -0.01 | 0.09 | 0.01 | -0.06 | 0.07 | -0.11 | -0.15 | 1 | 0.17 | 0.12 | -0.04 | -0.1 | -0.26* | 0.09 | 0.11 | 0.09 | -0.19 | -0.07 | 0.12 | 0.15 | 0.1 | 0.17 | -0.04 | 0.11 | 0.07 | -0.13 | 0.09 | -0.14 | -0.5** |
| Protein_powder | 0.18 | -0.03 | 0.09 | -0.02 | 0.03 | 0.08 | 0.02 | 0.03 | 0.17 | 1 | 0.36** | -0.09 | -0.08 | 0.05 | 0.3* | 0.15 | 0.43** | 0.05 | 0.06 | 0.13 | 0.12 | 0.13 | -0.09 | -0.01 | 0.13 | 0.08 | 0.06 | 0.03 | 0.02 | -0.01 |
| ENP | 0 | 0.16 | -0.18 | -0.22* | 0.09 | 0.13 | -0.05 | -0.04 | 0.12 | 0.36** | 1 | -0.06 | -0.04 | -0.03 | 0.07 | 0.13 | 0.33* | -0.09 | 0.04 | -0.06 | -0.05 | -0.08 | 0.15 | -0.14 | -0.08 | -0.16 | 0.18 | -0.12 | 0.05 | -0.11 |
| Sarcopenia_Pre | -0.08 | 0.04 | -0.49** | -0.45** | -0.01 | -0.21 | -0.04 | -0.17 | -0.04 | -0.09 | -0.06 | 1 | 0.33* | 0.2 | -0.4** | -0.03 | -0.14 | -0.17 | 0.03 | -0.39** | -0.39** | -0.39** | 0.09 | -0.15 | -0.39** | -0.49** | -0.14 | -0.14 | -0.19 | -0.06 |
| NRS2002_Cat | -0.12 | 0.28* | -0.34* | -0.31* | 0.14 | -0.04 | 0 | 0.02 | -0.1 | -0.08 | -0.04 | 0.33* | 1 | 0.45** | -0.28* | -0.32* | -0.37** | -0.25* | 0.11 | -0.31* | -0.3* | -0.33* | 0.22* | -0.08 | -0.32* | -0.37** | -0.32* | -0.04 | -0.11 | -0.07 |
| PG_SGA_Cat2 | -0.2 | 0.21* | -0.22* | -0.07 | 0.11 | -0.08 | -0.08 | -0.18 | -0.26* | 0.05 | -0.03 | 0.2 | 0.45** | 1 | -0.23* | -0.37** | -0.24* | -0.13 | 0.13 | -0.3* | -0.31* | -0.29* | 0.13 | 0.18 | -0.29* | -0.22* | -0.14 | -0.04 | -0.08 | 0.02 |
| Grip_strength | 0.66** | -0.44** | 0.5** | 0.2 | 0.05 | -0.13 | 0.32* | 0.39** | 0.09 | 0.3* | 0.07 | -0.4** | -0.28* | -0.23* | 1 | 0.3* | 0.45** | 0.54** | 0.08 | 0.68** | 0.66** | 0.69** | -0.42** | -0.28* | 0.68** | 0.54** | 0.39** | 0.28* | 0.07 | 0.29* |
| Energy | 0.47** | -0.22* | 0.2 | 0.03 | -0.13 | -0.14 | 0.44** | 0.31* | 0.11 | 0.15 | 0.13 | -0.03 | -0.32* | -0.37** | 0.3* | 1 | 0.76** | 0.38** | 0 | 0.44** | 0.43** | 0.44** | -0.31* | -0.32* | 0.44** | 0.3* | 0.27* | 0 | 0.15 | 0.19 |
| Protein | 0.39** | -0.25* | 0.25* | 0.11 | -0.01 | -0.07 | 0.31* | 0.2 | 0.09 | 0.43** | 0.33* | -0.14 | -0.37** | -0.24* | 0.45** | 0.76** | 1 | 0.41** | 0.13 | 0.39** | 0.36** | 0.4** | -0.35** | -0.18 | 0.39** | 0.34* | 0.31* | -0.04 | 0.1 | 0.23* |
| PhA | 0.56** | -0.5** | 0.34* | 0.31* | 0.04 | -0.18 | 0.34* | 0.45** | -0.19 | 0.05 | -0.09 | -0.17 | -0.25* | -0.13 | 0.54** | 0.38** | 0.41** | 1 | 0.18 | 0.43** | 0.35** | 0.49** | -0.91** | -0.23* | 0.44** | 0.54** | 0.48** | 0.32* | 0.27* | 0.57** |
| Delta_PhA_Cat_Mean | 0 | -0.08 | -0.11 | -0.11 | 0 | -0.05 | -0.07 | -0.13 | -0.07 | 0.06 | 0.04 | 0.03 | 0.11 | 0.13 | 0.08 | 0 | 0.13 | 0.18 | 1 | -0.03 | -0.05 | -0.01 | -0.17 | -0.07 | -0.02 | -0.01 | 0.18 | -0.02 | 0.06 | 0.02 |
| TBW | 0.65** | -0.3* | 0.8** | 0.46** | -0.06 | 0.05 | 0.28* | 0.37** | 0.12 | 0.13 | -0.06 | -0.39** | -0.31* | -0.3* | 0.68** | 0.44** | 0.39** | 0.43** | -0.03 | 1 | 0.99** | 1** | -0.29* | -0.21* | 1** | 0.78** | 0.33* | 0.27* | 0 | 0.27* |
| ECW | 0.64** | -0.26* | 0.78** | 0.43** | -0.07 | 0.07 | 0.27* | 0.34* | 0.15 | 0.12 | -0.05 | -0.39** | -0.3* | -0.31* | 0.66** | 0.43** | 0.36** | 0.35** | -0.05 | 0.99** | 1 | 0.98** | -0.19 | -0.22* | 0.99** | 0.75** | 0.3* | 0.26* | -0.02 | 0.22* |
| ICW | 0.66** | -0.33* | 0.8** | 0.47** | -0.05 | 0.03 | 0.29* | 0.38** | 0.1 | 0.13 | -0.08 | -0.39** | -0.33* | -0.29* | 0.69** | 0.44** | 0.4** | 0.49** | -0.01 | 1** | 0.98** | 1 | -0.35** | -0.21 | 1** | 0.8** | 0.36** | 0.28* | 0.01 | 0.31* |
| ECW_TBW_percent | -0.46** | 0.54** | -0.24* | -0.19 | -0.01 | 0.17 | -0.34* | -0.43** | 0.17 | -0.09 | 0.15 | 0.09 | 0.22* | 0.13 | -0.42** | -0.31* | -0.35** | -0.91** | -0.17 | -0.29* | -0.19 | -0.35** | 1 | 0.19 | -0.3* | -0.37** | -0.4** | -0.2 | -0.26* | -0.53** |
| PBF | -0.57** | 0.23* | 0.35** | 0.63** | 0.01 | 0.13 | -0.32* | -0.33* | -0.04 | -0.01 | -0.14 | -0.15 | -0.08 | 0.18 | -0.28* | -0.32* | -0.18 | -0.23* | -0.07 | -0.21* | -0.22* | -0.21 | 0.19 | 1 | -0.21* | 0.22* | -0.2 | -0.17 | -0.06 | -0.19 |
| BMR | 0.65** | -0.31* | 0.8** | 0.46** | -0.06 | 0.04 | 0.29* | 0.37** | 0.11 | 0.13 | -0.08 | -0.39** | -0.32* | -0.29* | 0.68** | 0.44** | 0.39** | 0.44** | -0.02 | 1** | 0.99** | 1** | -0.3* | -0.21* | 1 | 0.78** | 0.34** | 0.27* | 0 | 0.29* |
| AMC | 0.38** | -0.25* | 0.9** | 0.83** | -0.03 | 0.03 | 0.11 | 0.25* | 0.07 | 0.08 | -0.16 | -0.49** | -0.37** | -0.22* | 0.54** | 0.3* | 0.34* | 0.54** | -0.01 | 0.78** | 0.75** | 0.8** | -0.37** | 0.22* | 0.78** | 1 | 0.27* | 0.25* | 0.01 | 0.22* |
| PALB | 0.41** | -0.28* | 0.2 | 0.12 | 0.05 | -0.02 | 0.13 | 0.27* | -0.13 | 0.06 | 0.18 | -0.14 | -0.32* | -0.14 | 0.39** | 0.27* | 0.31* | 0.48** | 0.18 | 0.33* | 0.3* | 0.36** | -0.4** | -0.2 | 0.34** | 0.27* | 1 | 0.29* | 0.13 | 0.37** |
| Crea | 0.34* | -0.01 | 0.18 | 0.15 | -0.03 | -0.04 | 0.03 | 0.14 | 0.09 | 0.03 | -0.12 | -0.14 | -0.04 | -0.04 | 0.28* | 0 | -0.04 | 0.32* | -0.02 | 0.27* | 0.26* | 0.28* | -0.2 | -0.17 | 0.27* | 0.25* | 0.29* | 1 | 0.08 | 0.08 |
| WBC | 0.09 | -0.08 | 0.01 | 0.03 | -0.01 | 0.12 | 0.27* | 0.3* | -0.14 | 0.02 | 0.05 | -0.19 | -0.11 | -0.08 | 0.07 | 0.15 | 0.1 | 0.27* | 0.06 | 0 | -0.02 | 0.01 | -0.26* | -0.06 | 0 | 0.01 | 0.13 | 0.08 | 1 | 0.22* |
| HGB | 0.37** | -0.25* | 0.16 | 0.07 | 0.11 | -0.22* | 0.28* | 0.29* | -0.5** | -0.01 | -0.11 | -0.06 | -0.07 | 0.02 | 0.29* | 0.19 | 0.23* | 0.57** | 0.02 | 0.27* | 0.22* | 0.31* | -0.53** | -0.19 | 0.29* | 0.22* | 0.37** | 0.08 | 0.22* | 1 |

Note: ΔPA_Cat, categorized perioperative phase angle decline (dichotomized at the cohort mean of 0.32°); AMC, arm muscle circumference; ALB, albumin; BCM, body cell mass; BMR, basal metabolic rate; BMI, body mass index; Crea, serum creatinine; ECW, extracellular water; ECW/TBW (%), percentage of extracellular water to total body water ratio; FFMI, fat-free mass index; HGB, haemoglobin; ICW, intracellular water; NRS2002, Nutritional Risk Screening 2002; PA, phase angle; PALB, prealbumin; PBF%, percentage body fat; PG-SGA, patient-generated subjective global assessment; TBW, total body water; WBC, white blood cell. * *p* < 0.05, ** *p* < 0.001.

## **TABLE S3.** Bootstrap and ridge regression validation of variables for postoperative sarcopenia (N = 90).

| **Variable** | **Bootstrap CV (%)** | **Ridge Coefficient (λ_min)** | **Ridge Coefficient (λ_1se)** |
| --- | --- | --- | --- |
| ΔPhA_Cat (High vs. Low) | 109 | 0.74 | 0.25 |
| NRS2002_Cat (≥3) | 78.9 | 1.1 | 0.54 |
| Sarcopenia_Pre (Yes) | 86 | 1.36 | 0.67 |
| BMI (kg/m²) | 101.4 | –0.16 | –0.06 |
| AMC (cm) | 293.8 | –0.16 | –0.08 |
| ECW/TBW (%) | 177.6 | 0.3 | 0.12 |

Note: Low ΔPhA, phase angle decline < 0.32°; High ΔPhA, phase angle decline ≥ 0.32°; AMC, arm muscle circumference; ECW/TBW (%), percentage of extracellular water to total body water ratio; CV, coefficient of variation; λ_min, optimal penalization; λ_1se, conservative penalization.

## **TABLE S4.** Sensitivity analysis treating ΔPhA as a continuous variable (N = 90).

| **Variable** | **OR** | **95% CI** | ***p* value** |
| --- | --- | --- | --- |
| ΔPhA, per 0.1° increase | 1.09 | (0.90–1.32) | 0.387 |
| Sarcopenia_Pre (Yes vs. No) | 14.69 | (3.02–87.53) | 0.001 |
| NRS2002_Cat (≥3 vs. <3) | 9.41 | (2.06–46.44) | 0.004 |

Note: Model adjusted for ΔPhA (continuous), preoperative sarcopenia, and nutritional risk (NRS2002_Cat). ΔPhA is expressed per 0.1° increase for clinical interpretability. NRS2002_Cat, dichotomized NRS2002 as ≥ 3 or < 3; Sarcopenia_Pre, preoperative sarcopenia.

## **TABLE S5.** Sensitivity analysis using Firth penalized logistic regression for postoperative sarcopenia (N = 90).

| **Variable** | **Standard Logistic Regression** | | **Firth Penalized Regression** | |
| --- | --- | --- | --- | --- |
|  | OR (95% CI) | *p* value | OR (95% CI) | *p* value |
| ΔPhA_Cat (High vs. Low) | 4.70 (1.17–24.39) | 0.040 | 3.95 (1.08–17.59) | 0.037 |
| NRS2002_Cat (≥3 vs. <3) | 8.57 (1.76–46.22) | 0.008 | 7.37 (1.65–35.55) | 0.009 |
| Sarcopenia_Pre (Yes vs. No) | 21.57 (3.90–163.70) | < 0.001 | 16.18 (3.31–103.10) | < 0.001 |

Note: Low ΔPhA, phase angle decline < 0.32°; High ΔPhA, phase angle decline ≥ 0.32°; NRS2002_Cat, dichotomized NRS2002 as ≥ 3 or < 3; Sarcopenia_Pre, preoperative sarcopenia.
